# Research on Embodied Carbon Transfer Measurement and Carbon Compensation among Regions in China

**DOI:** 10.3390/ijerph20032761

**Published:** 2023-02-03

**Authors:** Hao Chen, Erdan Wang, Nuo Wang, Tao Song

**Affiliations:** 1 School of Economics and Resource Management, Beijing Normal University, Beijing 100875, China; 2 Beijing Key Lab of Study on SCI-TECH Strategy for Urban Green Development, Beijing 100875, China

**Keywords:** embodied carbon, net carbon transfer, spatial layout, carbon compensation, multiregional input–output model

## Abstract

The existence of interprovincial embodied carbon transfer not only makes it difficult to achieve carbon emission reductions but also exacerbates the inequity, inefficiency, and high costs of interprovincial carbon emission reduction rights and responsibilities. This paper uses multi-regional input–output analysis (MRIOA) to measure the interprovincial embodied carbon transfer in 2017, obtains the net carbon transfer between 30 provinces (municipalities and autonomous regions) and eight regions in 2017, and accounts for the interprovincial carbon compensation amount based on the carbon price in the national carbon market. This study finds that carbon transfer from economically developed provinces to less developed provinces still exists in China, and the overall distribution shows a spatial transfer pattern from south to north and from east to west, with the northwestern region bearing most of the carbon emission pressure for which it should receive corresponding financial compensation. As part of the process to achieve the “dual carbon” target, appropriate emission reduction policies should be formulated according to the characteristics of provincial carbon transfer and the principle of “who benefits, who compensates”, and economically developed regions should give corresponding financial or technical compensation to less developed regions based on net carbon transfer. Compensation and support should be given to less developed regions based on net carbon transfer to prevent further regional development imbalances.

## 1. Introduction

In 2020, China pledged to the international community that it would strive for a peak in carbon emissions by 2030 and work toward achieving carbon neutrality by 2060 (http://www.xinhuanet.com/ (accessed on 15 December 2022)). To realize this goal, all sectors of society have actively contributed ideas and solutions, relevant enterprises have quickly released their carbon peaking and carbon-neutral plans, and various provinces (municipalities and autonomous regions) have included relevant action plans in their government work reports.

China is a major emitter of carbon dioxide, and the IEA statistical report shows that China’s CO_2_ emissions accounted for33% (https://www.iea.org/reports/global-energy-review-co2-emissions-in-2021-2 (accessed on 15 December 2022)) of the total global CO_2_ emissions in 2021 There is no doubt that China is under great pressure to reduce carbon dioxide emissions. Furthermore, to achieve the dual carbon target, it is necessary to decompose and implement the responsibility of carbon emission reduction to each province and region. However, China is a vast country with large differences in geographic distributions, resource patterns, industrial structures, and economic development levels among regions, and these regions have strong spatial correlations. The extensive and close economic and trade ties between provinces and regions may lead to a spatial transfer of carbon emissions, thus affecting the total carbon emissions of each province. Economically developed regions transfer high-carbon-emission products from less developed provincial regions through inter-regional trade. The less developed provincial regions produce such products for a long time based on their resource endowment and industrial base for the sake of economic development. This results in the phenomenon that although the developed regions are effective in reducing emissions, but it aggravates the pressure of carbon emission reduction in less developed regions. Scholars have conducted a series of studies on this situation and found that there is a carbon transfer pattern in inter-regional trade, which is referred to as embodied carbon [1,2,3,4,5,6].

Research shows that the inter-regional transfer of carbon emissions has directly led to an inequity in carbon emission reduction rights and responsibilities among regions, instead of achieving reductions in overall carbon emissions [7,8]. Embodied inter-regional carbon transfers have a significant impact on regional carbon emission patterns and carbon emission reductions. The resulting transfer of inter-regional emission reduction pressure is important as it offsets the overall emission reduction effect, thereby increasing the burden of economic growth for net carbon emission transfer areas and the difficulties in upgrading and transforming industrial structures. Meanwhile, practices show that to ensure China’s rapid economic development and achieve the emission reduction targets set by the Chinese government and interprovincial regions, it is necessary to accurately grasp the characteristics of regional carbon emission spatial transfer, clarify its economic spillover effects, and effectively guide the reasonable transfer of carbon emissions among interprovincial regions in China. These tasks represent the starting point of this paper.

With the marketization and regional integration process in China, economic ties between provinces and regions have become increasingly close and trade in goods and services has become more frequent. The carbon embodied in the inter-regional trade of goods and services has led to discrepancies between the carbon footprints of provinces and regions and the actual carbon emissions. Therefore, it is important to consider the spatial transfer of carbon emissions for inter-regional cooperation on energy and emission reduction. This paper mainly discusses two problems: (1) establishing the characteristics of interprovincial flows of carbon in China (regions and sectors) and (2) conducting regional carbon compensation. Therefore, this paper adopts multi-regional input–output analysis (MRIOA), based on the latest multi-regional input–output data, to measure the embodied carbon transfer levels of 29 industries in 30 provinces (municipalities and autonomous regions) in 2017 and analyzes the specific value and spatial distribution pattern of the embodied carbon transfer between provinces and regions in China, as well as the compensation policies between regions. This paper aims to explore the interprovincial carbon transfer structure and clarify the different roles of regions with closely related economies and significantly different carbon emission patterns in the emission reduction process. It is of great theoretical and practical significance to promote the formation of inter-regional synergy and formulate the scientific and rational carbon emission reduction policies in each province and region.

## 2. Literature Review

### 2.1. Embodied Carbon Transfer

Since the 1990s, international trade has achieved rapid growth with the wave of economic globalization. International trade is based on domestic production and foreign demand, and the consumption of fossil energy in the production process causes greenhouse gas emissions [9,10,11]. As the impact of CO_2_ emissions on global warming has attracted strong global attention, global cooperation on carbon emission reduction has deepened, and the game between regions in areas such as carbon emission responsibility and reduction targets has become increasingly intense [3]. Carbon dioxide emissions were found to be increased by some socioeconomic factors such as urbanization, population density, economic growth, and fossil fuels [12,13]. Distinguishing the share of carbon emissions and the division of emission reduction responsibilities among trading partners has an important impact on the cooperation among countries to cope with global climate change. Thus, the embodied carbon transferred among countries has become a research hotspot.

Scholars have found that economically developed countries themselves emit little carbon, but their trade-induced correlated energy consumption and carbon emissions can be significant [14,15,16]. Based on this, scholars have conducted a lot of research on the issue of embodied carbon emission transfer in international trade, from the perspective of individual countries or regions [1,2,3,6], or a comprehensive exploration of the issue of embodied carbon in trade between major global countries or regions, with a broader scope of research [17,18,19,20]. On the one hand, from the perspective of the amount and direction of embodied carbon transfer, the embodied carbon in global trade is rapidly increasing, and the embodied carbon in trade of goods and services amounted to 22% of the total global carbon emissions in 2004, and the embodied carbon in global trade accounted for 1/4 of the total global carbon emissions in 2012 [21]. On the other hand, from a geospatial perspective, consumers and producers of pollutants (e.g., CO_2_ emissions) in international trade are separated geospatially. One country can transfer its consumption-related pollutant emissions to other countries or regions through international trade, which also transfers part of the carbon emission reduction obligations originally belonging to consumers and producers [15,22]. From the perspective of both global and regional development, the impact of embodied carbon transfer on regional carbon emissions is not only extensive, long-lasting, and deep-routed, but also plays an important role that must be considered in the study of regional carbon emissions.

Scholars have identified a possible carbon reduction inequity among Chinese provinces (municipalities and autonomous regions) that are undertaking carbon reduction actions [23,24,25,26]. Some energy-rich provinces, such as Inner Mongolia, Shanxi, and Hebei [27,28,29,30], have long supplied energy-intensive products to other provinces, which have become carbon-emission-importing provinces. Guangdong, Jiangsu, Zhejiang, and other areas have developed manufacturing industries. To maintain their economic growth, they need to purchase large amounts of energy, raw materials, and end consumer goods from other provinces, which is the equivalent of transferring part of their carbon emissions through the consumption of high-carbon products produced in other provinces. Therefore, the energy-intensive regions in the central and western regions invariably bear a portion of the carbon emissions from other provinces. They are at a disadvantage in terms of the spatial allocation and economic benefits of carbon emissions. The embodied various carbon study methods, mainly including the life cycle approach (LCA), the consumer lifestyle approach, and the input–output approach [1,14,31,32]. Since the first two methods have high data requirements when applied, data collection requires a lot of time, money, labor, etc., and data selection is conducted manually which may lead to impractical analysis, while the input–output analysis method is more commonly applied in comparison to the life cycle method and the consumer lifestyle method [5,33,34]. The multi-regional input–output analysis links different regions and enables the study of the various linkages that exist between them. With the development and improvement of multi-regional input–output database, more and more scholars use multi-regional input–output analysis to research environmental problems, such as energy footprints and carbon emissions [35,36], water consumption [37,38,39], and biodiversity [6,40].

Overall, previous studies have helped to clarify the characteristics and division of responsibilities for embodied carbon transfers between countries or regions. Our study differs in the way in which we focus on interprovincial and eight regions embodied carbon spatial transfer characteristics (carbon transfer-out levels, carbon transfer-in levels, and net carbon transfer levels) and identifying the key sectors of carbon transfer at the provincial level. Based on carbon transfer measurement data, we propose a specific accounting scheme for regional horizontal carbon compensation. Meanwhile, current research on interprovincial embodied carbon transfer is mainly based on the input–output table developed before 2012 to measure interprovincial carbon transfer. With the rapid development of the economy, the provinces have adjusted the industrial structure and technology, and the interprovincial trade structure has also changed. These new developments demand the remeasurement and reanalysis of interprovincial embodied carbon transfer in time and space, which are necessary for an in-depth study of the embodied carbon problem. Based on this, we measured the aggregate characteristics and spatial patterns of inter-provincial carbon emission transfers in China using the latest data on China’s inter-regional inputs and outputs.

### 2.2. Carbon Compensation

In response to inequitable carbon emission reductions between regions, scholars have begun exploring the implementation of inter-provincial financial or technical compensation to ensure fairness in regional carbon emission reduction cooperation. Research on carbon compensation can be traced to studies on the coordination and compensation of resource interests between regions and ecological compensation. Payments for ecosystem/environmental services (PESs) aim to protect the ecological environment and promote the harmonious development of humankind and nature. By internalizing the economic externalities in ecological protection, PESs adopt public policy instruments or market-based instruments to adjust the relationship between ecological protection areas and ecologically beneficial areas. Ecological compensation should first clarify the environmental service provider (recipient) and the compensation payer (compensator) [41,42], and then determine the standard of ecological compensation [43,44,45]. Carbon compensation is a new field of ecological compensation research designed to address global climate change and develop a low-carbon economy. International research includes forest carbon compensation [46,47], carbon compensation technologies [48], and regional carbon emission allowance allocation mechanisms [49,50]. Meanwhile, other exploratory studies (Zhao, Rongqin et al., (2015) [51]; Zhang, Wei (2019) [52]; and Wan, Lunlai et al., (2020) [53]) have conducted a more systematic and elaborate study on the characteristics of carbon compensation, which includes regional carbon compensation and a basic framework for a carbon trading system.

Compensation can be divided into vertical compensation and horizontal compensation. Vertical ecological compensation with administrative affiliation between the compensator and the recipient entails the ecological compensation carried out by both the central government and the local government at different levels, as well as the provincial government in relation to cities, counties, townships, etc., within an administrative jurisdiction. Horizontal ecological compensation refers to those without administrative affiliation between the compensator and the recipient, such as interprovincial or intermunicipal ecological compensation [54,55]. Examples include compensation for resource derivatives and market transactions such as resource use rights, emission rights, and carbon emission rights. The methods include interprovincial horizontal financial transfer payments between cities and counties, the joint construction of industrial parks, and horizontal compensation through funds. The method also includes technical support for marginal regions from economic development regions, which can help marginal regions achieve energy conservation and emission reduction, while supporting economic development regions in achieving their own carbon emission targets. Regional horizontal carbon compensation is conducive to promoting urban–rural integration and narrowing the gap between regional economic and social development.

Regional horizontal carbon compensation represents an important means of achieving regional equitable development. Regional horizontal carbon compensation mainly includes three approaches [53,56,57]: (1) direct economic compensation; (2) technical support; and (3) a regional carbon emission quota system. This paper adopts regional horizontal economic compensation, based on inter-provincial net carbon transfer volumes. A certain amount of economic compensation is given by the net carbon transfer-out region to the net carbon transfer-in region to promote regional equitable development.

## 3. Methodology and Data

### 3.1. MRIOA

**Figure 1:**
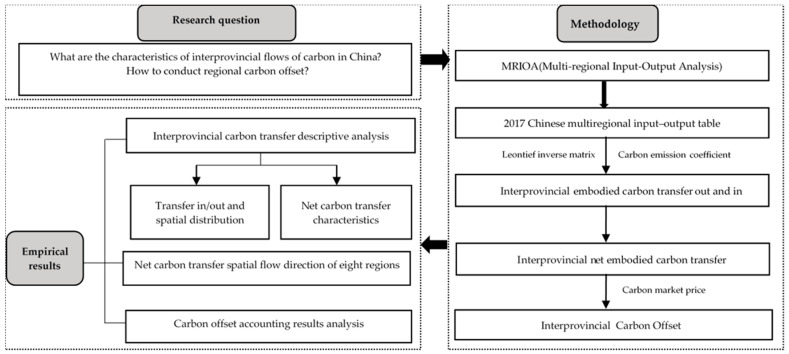
Research flowchart of this study. Source: the authors.

As shown in Figure 1, this study evaluated the characteristics of inter-regional carbon transfer and proposed carbon compensation measures based on multi-regional input–output analysis.

Multi-regional input–output analysis (MRIOA) is based on multi-regional input–output tables with the Leontief inverse matrix as the core, which can accurately quantify the economic linkages between regions and sectors. Then, combined with the carbon emission coefficient of the sector, the carbon emissions and carbon transfer can be accurately calculated. The multi-regional input–output table is shown in Table 1.

The equilibrium equation of the value-based inter-regional input–output model is:

(1) $$X_{i}^{p}=\sum_{q=1}^{m}\sum_{p=1}^{n\;\;}X_{ij}^{pq}+\sum_{q=1}^{m}Y_{i}^{pq}+EX_{i}^{p}$$

The superscript denotes the region and the subscript denotes the sector. $X_{ij}^{pq}$ denotes the intermediate input of sector *i* in region *p* to sector *j* in region *q*, $Y_{i}^{pq}$ denotes the final product provided by sector *i* in region *p* to region *q*, $EX_{i}^{p}$ denotes the export of sector *i* in region *p*, and $X_{i}^{p}$ denotes the total output of sector *i* in region *p* (where *p*, *q* = 1, 2, …, *m* and *I*, *j* = 1, 2, …, *n*).

Let $a_{ij}^{pq}=\frac{X_{ij}^{pq}}{X_{j}^{q}}$, $a_{ij}^{pq}$ be the direct consumption coefficient.

Let $A^{pq}=\left[\begin{array}{cccc} a_{11}^{pq} & a_{12}^{pq} & \cdots & a_{1n}^{pq}\\ a_{21}^{pq} & a_{22}^{pq} & \cdots & a_{2n}^{pq}\\ \vdots & \vdots & \ddots & \vdots \\ a_{n1}^{pq} & a_{n2}^{pq} & \ldots & a_{nn}^{pq} \end{array}\right]$ and $A^{pq}$ denote the subregional direct consumption coefficient matrices, i.e., the direct consumption coefficient matrix of intermediate product inputs from region *p* to region *q*.

Therefore, Equation (1) can be written in matrix form, as shown below.

(2) $$\left[\begin{array}{c} X^{1}\\ X^{2}\\ \vdots \\ X^{m} \end{array}\right]=\left[\begin{array}{cccc} A^{11} & A^{12} & \cdots & A^{1m}\\ A^{21} & A^{22} & \cdots & A^{2m}\\ \vdots & \vdots & \ddots & \vdots \\ A^{m1} & A^{m2} & \cdots & A^{mm} \end{array}\right]\left[\begin{array}{c} X^{1}\\ X^{2}\\ \vdots \\ X^{m} \end{array}\right]+\left[\begin{array}{ccccccccc} Y^{11} & + & Y^{12} & + & \cdots & + & Y^{1m} & + & {EX}^{1}\\ Y^{21} & + & Y^{22} & + & \cdots & + & Y^{2m} & + & {EX}^{2}\\ \vdots & \vdots & \vdots & \vdots & \cdots & \vdots & \vdots & \vdots & \vdots \\ Y^{m1} & + & Y^{m2} & + & ∆ & + & Y^{mm} & + & {EX}^{m} \end{array}\right]$$

In the above matrix, $X^{p}$ denotes the column vector of the total output of each sector in region *p*, $Y^{pq}$ denotes the column vector of products produced in region *p* as end-use products in region *q*, and $EX^{p}$ is the column vector of products exported from region *p*. It is further found that:

(3) $$X^{p}={\left(I-A^{pp}\right)}^{-1}\left(\sum_{\underset{p\neq q}{q=1}}^{m}A^{pq}X^{q}+\sum_{\underset{p\neq q}{q=1}}^{m}Y^{pq}+Y^{pp}+{EX}^{p}\right)$$

In the above equation, ${\left(I-A^{pp}\right)}^{-1}$ is the inter-regional Leontief inverse matrix, ${\left(I-A^{pp}\right)}^{-1}\sum_{\underset{p\neq q}{q=1}}^{m}A^{pq}X^{q}$ denotes the total output of region *p* to meet the needs of intermediate products in other regions, ${\left(I-A^{pp}\right)}^{-1}\sum_{\underset{p\neq q}{q=1}}^{m}Y^{pq}$ denotes the total output of region *p* to meet the needs of final products in other regions, ${\left(I-A^{pp}\right)}^{-1}Y^{pp}$ denotes the total output of region *p* to meet the final product needs of this region, and ${\left(I-A^{pp}\right)}^{-1}EX^{p}$ denotes the total output of region *p* to meet export needs.

### 3.2. Carbon Transfer Measurement Method

The introduction of carbon emission coefficient shifts the value-based flow relationship between regions to a carbon emission flow relationship. The carbon emission coefficient ($c_{i}$) refers to the carbon emissions per unit of output in the sector.

Let $C^{p}$ be the diagonal matrix with CO_2_ emission coefficient $c_{i}$ (*i* = 29) for each industrial sector in region *p*; then, the column vector of CO_2_ emissions for each industrial sector in region *p* is shown below:

(4) $$C^{p}X^{p}=C^{p}{\left(I-A^{pp}\right)}^{-1}\left(\sum_{\underset{p\neq q}{q=1}}^{m}A^{pq}X^{q}+\sum_{\underset{p\neq q}{q=1}}^{m}Y^{pq}+Y^{pp}+EX^{p}\right)$$

In the above equation, $C^{p}{\left(I-A^{pp}\right)}^{-1}\sum_{\underset{p\neq q}{q=1}}^{m}A^{pq}X^{q}$ is the amount of CO_2_ emitted in region *p* to meet the demand for intermediate products in other regions; $C^{p}{\left(I-A^{pp}\right)}^{-1}\sum_{\underset{p\neq q}{q=1}}^{m}Y^{pq}\;\mathrm{and}\;C^{p}{\left(I-A^{pp}\right)}^{-1}Y^{pp}$ denote the amount of CO_2_ emitted in region *p* to meet the demand for final products in other regions and in its own; and $C^{p}{\left(I-A^{pp}\right)}^{-1}EX^{p}$ is the amount of CO_2_ emitted in the *p* region to meet exports.

For two regions, the net carbon transfer between each other is $nct_{p}^{q}$, which represents the difference between the total amount of embodied carbon transferred from region *p* to region *q* (transfer-out amount) and the total amount of embodied carbon transferred from *q* to region *p* (transfer-in amount).

(5) $$nct_{p}^{q}=C^{q}{\left(I-A^{qq}\right)}^{-1}\left(A^{qp}X^{p}+Y^{qp}\right)-C^{p}{\left(I-A^{pp}\right)}^{-1}\left(A^{pq}X^{q}+Y^{pq}\right)$$

If $nct_{p}^{q}$ > 0, it means that region *p* transferred carbon to region *q*. If $nct_{p}^{q}$ < 0, it means that region *q* transferred carbon emissions to region *p*.

The net carbon transfer (NCT) represents the difference between the total amount of embodied carbon transferred from region *p* to other regions (transfer out) and the total amount of embodied carbon transferred from other regions to region *p* (transfer in). The equation for the net carbon transfer from region *p* is shown below:

(6) $${\mathrm{NCT}}_{p}=\sum_{\underset{q\neq p}{q=1}}^{\mathrm{30}}c^{q}{\left(I-A^{qq}\right)}^{-1}\left(A^{qp}X^{p}+Y^{qp}\right)-\sum_{\underset{q\neq p}{q=1}}^{\mathrm{30}}c^{p}{\left(I-A^{pp}\right)}^{-1}\left(A^{pq}X^{q}+Y^{pq}\right)$$

If ${\mathrm{NCT}}_{p}$ > 0, it means that region *p* transferred carbon to other provinces. If ${\mathrm{NCT}}_{p}$ < 0, it means that all other provinces transferred carbon emissions to region *p*.

### 3.3. Carbon Offset Measurement Approach

For carbon compensation accounting, the horizontal compensation method is adopted. First, the recipient and the compensator have a clear agreement on their respective responsibilities and rights. This paper divides the responsibilities and rights of both the recipient and the compensator based on the amount of net carbon transfer. The net carbon transfer-in region is the recipient and the net carbon transfer-out region is the compensator. Second, compensation funds should be accounted for with certain criteria. In carbon compensation accounting, the recipient party provides intermediate use and final consumption products for the compensator but increases its ecological protection and environmental management costs. Therefore, the compensator needs to provide compensation funds to the recipient.

Accounting of compensation funds is based on the carbon emission transaction price. On 16 July 2021, the national carbon emission trading market was officially launched; the trading center is located in Shanghai. The average transaction price of RMB 51.23 per ton on the first day of the national carbon market represents a continuation in the improvement of China’s carbon pricing mechanism, as the carbon market is expected to grow with an annual trading volume of over RMB 100 billion, providing price signals as well as financial support for the carbon reduction actions of the whole society. Accounting for carbon compensation on this basis can more accurately reflect the current value of carbon. Therefore, this paper selects an average price of 51.23 RMB/ton (http://www.xinhuanet.com/ (accessed on 15 December 2022)) of equivalent CO_2_ on the first day of trading and converts carbon transfer into an amount to determine the interprovincial compensation amount.

(7) $${\mathrm{CA}}_{p}^{q}=nct_{p}^{q}\times P$$

In the above equation, ${\mathrm{CA}}_{p}^{q}$ denotes the amount of carbon compensation that region *p* needs to give to region *q*. $nct_{p}^{q}$ denotes the net carbon transfer from region *p* to region *q*. P = 51.23. If ${\mathrm{CA}}_{p}^{q}$ > 0, it means that region *p* needs to compensate for region *q*. If ${\mathrm{CA}}_{p}^{q}$ < 0, it means that region *q* needs to compensate for region *p*.

### 3.4. Data Sources

In this paper, China’s multiregional input–output tables and CO_2_ emission factors for each region and sector were required to calculate the CO_2_ transfers among 30 provinces and between sectors. The Chinese multiregional input–output data were selected from the 2017 Chinese multiregional input–output table compiled by Zheng et al., (2020) [34], which is updated every 5 years for each province in China; these data are the latest Chinese multiregional input–output data for the value of trade flows among the 30 provinces and 42 sectors.

There is no official published platform in China for CO_2_ emission factors by region and sector, so this study uses the carbon emission inventory of each province compiled by the China Carbon Accounting Database (CEAD) (https://www.ceads.net/data/ (accessed on 15 December 2022)) based on the IPCC sectoral carbon accounting methodology and the 2017 China Coal Industry Yearbook. This emission inventory includes energy carbon emissions from the combustion of 17 fossil fuels and process carbon emissions from the production of cement in order to measure carbon emissions from 45 sectors using carbon emission factors consistent with Chinese data. To maintain data consistency, we organized the carbon emission inventory into 29 sectors and accordingly consolidated the multiregional input–output tables into 29 sectors for 30 regions.

## 4. Empirical Results

### 4.1. Interprovincial Carbon Transfer Accounting Analysis

#### 4.1.1. Analysis of Carbon Transfer Characteristics and Spatial Distribution

**Figure 2:**
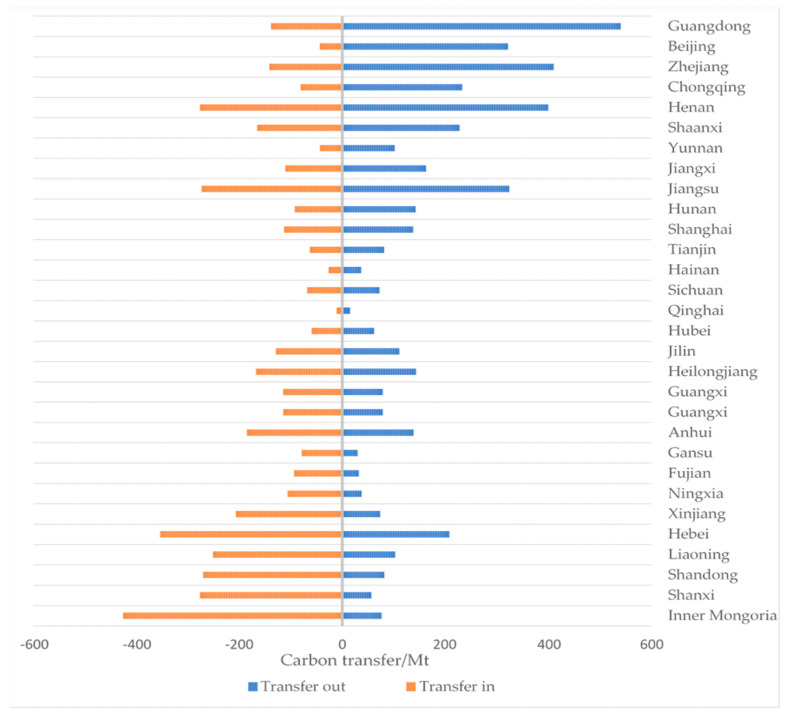
Carbon transfer from 30 provinces (municipalities and autonomous regions).

The net carbon transfer of each province (municipalities and autonomous regions) was calculated. Transfers in carbon emissions are those generated by one region to meet the needs of other regions, or generated by other regions as consumer goods for their own production activities or final consumption; the converse are called the transfer-out regions. The results of the 30 provinces (municipalities and autonomous regions) are shown in Figure 2. 

From a provincial perspective, as shown in Figure 2, the province with the most carbon transfer-out regions is Guangdong Province, followed by Henan, Zhejiang, and Jiangsu; the province with the most carbon transfer-in regions is the Inner Mongolia Autonomous Region, followed by Hebei, Henan, and Shanxi. From a sectoral perspective, 80–90% of carbon transfer out from the top four provinces comes from electricity and hot water production, metallurgy, nonmetal products, coal mining, petroleum, and transportation industry. Among them, electricity and hot water production belongs to the energy industry and the transportation industry belongs to the service industry. Accordingly, in terms of carbon transfer in sectors, 80–90% of carbon transfer-in levels from the top four provinces also come from these types of sectors. In other provinces, the sectors with the highest carbon transfer-out and transfer-in levels are also concentrated in the energy sector, petrochemical and mineral sectors, and the transportation sector. There is no doubt that these sectors belong to high-carbon-emission sectors. By improving the energy structure, energy efficiency, and carbon emission efficiency, the goal of carbon emission reduction can be gradually achieved.

**Figure 3:**
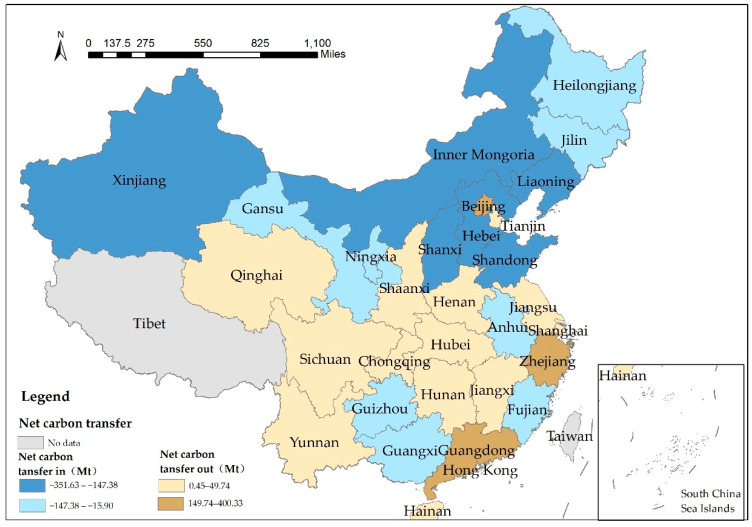
Spatial distribution of net carbon transfer by region in 2017.

The spatial distribution of net carbon transfer-in levels and net carbon transfer-out levels of each province (municipalities and autonomous regions) is shown in the map of China using ArcGIS10.3.1 (Figure 3).

It can be seen that most of the net carbon transfer levels in areas are distributed in the north with a few areas in the south, while most of the net carbon transfer out areas are distributed in Beijing and Tianjin, as well as central and eastern coastal areas. The overall distribution shows a spatial shifting pattern from south to north and from east to west. This spatial distribution pattern illustrates the overall presentation of carbon transfer from economically developed provinces to less developed provinces. It can be seen from the 2017 China Coal Industry Yearbook that Beijing, Shanghai, Jiangsu, Henan, Guangdong, and other regions are the net transfer-in regions of coal from other provinces in 2017, while Inner Mongolia, Shanxi, Shaanxi, Xinjiang, and other places are the net transfer-out regions of coal. Based on natural resource endowment, the economic development of these provinces relies on local natural resources; energy demand has rigid characteristics, and most of the energy-consuming products of high-energy-consuming industries are designed to meet the consumption of other regions. Meanwhile, in the more economically developed regions, such as North China, Shanghai, and Guangzhou, tertiary industries account for more than half of the total industries; when coupled with the environmental regulation requirements, these regions are motivated to obtain energy-intensive products from other regions to meet production and consumer needs, leading to low local carbon emissions.

Thus, the provinces with more carbon transfer-out levels import much more energy from less developed but energy-rich provinces to meet their production and consumption demands, and while the carbon emissions produced by them are very small, their consumers are responsible for high levels of carbon emissions. Most resource-based provinces, such as Inner Mongolia, provide many energy products to economically developed provinces, such as Beijing and Guangdong. The development of interprovincial trade has led to an increase in the volume of products moving between provinces; the locations of production and consumption are different, which leads to inter-regional carbon transfer.

#### 4.1.2. Analysis of Inter-Provincial Net Carbon Transfer Characteristics

**Figure 4:**
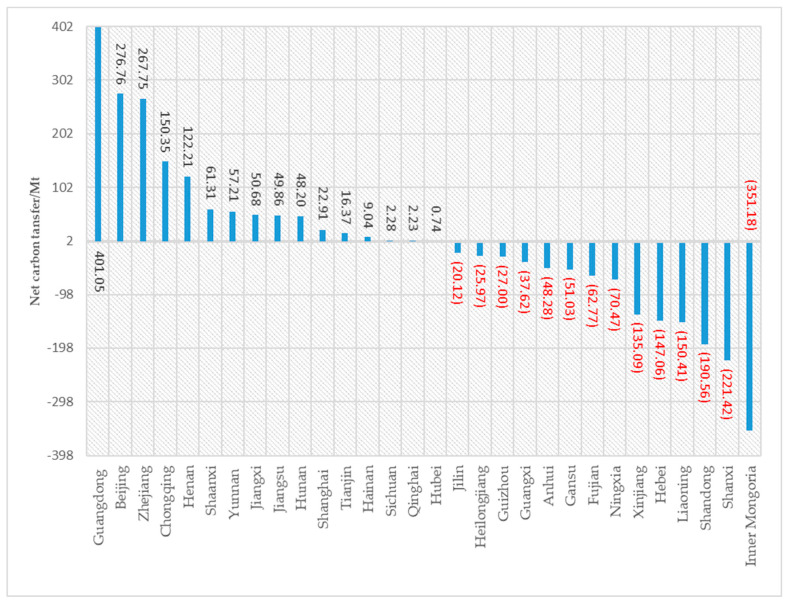
Net carbon transfer of provinces (cities and autonomous regions) in 2017.

The net carbon transfer is obtained by subtracting the carbon transfer-out levels from the carbon transfer-in levels (Figure 4). The 16 provinces of Beijing, Tianjin, Shanghai, Jiangsu, Zhejiang, Jiangxi, Henan, Hunan, Hubei, Guangdong, Hainan, Chongqing, Sichuan, Yunnan, Shaanxi, and Qinghai have a positive net carbon transfer, while the remaining 14 provinces have a negative net carbon transfer. The research results indicate that there is a “carbon leakage” between economically developed provinces and less developed provinces within China. The 16 provinces with positive net carbon transfer are all more economically developed regions. Among them, Guangdong, Beijing, Zhejiang, Zhejiang, Henan, and Chongqing have large net carbon transfer-out levels, of which the largest is Guangdong with 400 million tons. These regions either have a single industrial structure or an imperfect industrial structure, and have to rely on imported materials to meet final demands. Among the 14 provinces with negative net carbon transfer levels, Inner Mongolia, Shanxi, Xinjiang, Shandong, Hebei, and Jilin have large net carbon transfer-in amounts, with the largest net carbon transfer occurring in Inner Mongolia at 350 million tons. Taking Inner Mongolia region as an example, the largest sector of net carbon transfer is the electricity and hot water production (energy sector). Inner Mongolia is an important energy base. In its primary energy production structure, raw coal accounts for more than 90%. Its energy production not only serves its own economic development, but also indirectly supports the economic growth of other provinces through inter-provincial trade. From a sectoral perspective, the net carbon transfer in Inner Mongolia, Shanxi, Shandong, and Ningxia mainly comes from the energy sector, which should focus on improving the efficiency of energy use and changing the energy structure. While the net carbon transfer in Hebei and Liaoning mainly comes from the nonmetal and metal sectors, which should reduce carbon from three aspects: energy structure, low-carbon technology (mining technology, mineral processing technology, etc.), and management (production process, transportation, etc.).

### 4.2. Analysis of the Net Carbon Spatial Transfer Characteristics of Eight Regions

The National Information Center divided China’s mainland into eight regions defined by precise regional policies and spatial segment subdivisions. Based on the input–output tables of the regions, the similarity of industrial structures and economic development levels of provinces (regions) and cities, as well as their geographical relationships. Many scholars have discussed the spatial association of regional economies and their dynamic evolution characteristics on this basis [5,58,59,60]. The classification and net carbon transfer of the eight regions are shown in Table 2.

From the values of regional net carbon transfer, we can see that four regions have a positive net carbon transfer, which means that other regions bear the corresponding carbon emission pressure values for the regions. These regions are the Northern Metropolis, the Central Coast Region, the Southern Coast Region, and the Southwest Region, which have less energy-intensive industries and more developed low-energy industries and services in their economic structure or have imperfect economic structures, and are more dependent on imports from other resource-based regions for energy and other products. The remaining four regions have a negative net transfer, indicating that they bear part of the carbon emission pressure for other regions. These regions are the northern coastal Region, the northeast region, the central region, and the northwest region. The four regions are natural-resource-rich regions, and especially bare lots of energy, including raw coal, crude oil, primary electricity, etc. These provide means of production for production activities in other regions through the exportation of energy products, the flow of products in the intermediate sector, and the trans-regional transmission of electricity (likewise bearing corresponding carbon emissions). The net transfer of regional carbon emissions reflects their status and role in the national regional pattern of carbon emission reduction.

**Figure 5:**
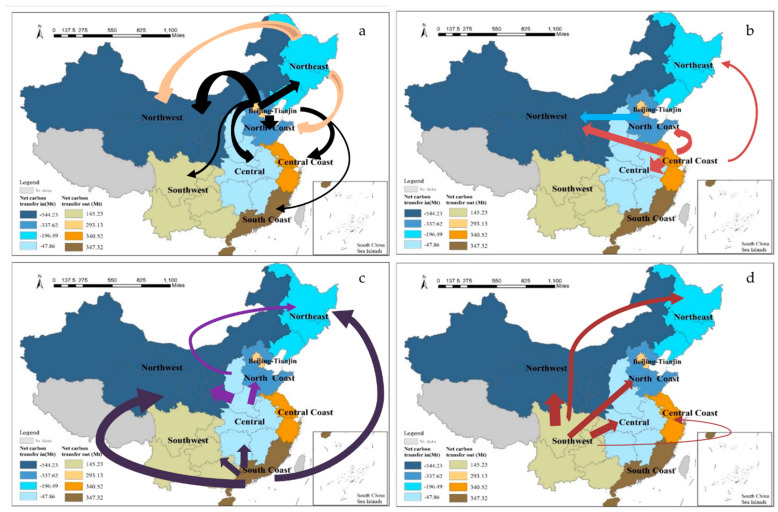
Spatial distribution and steering of net carbon transfer in the eight regions. (**a**) Net carbon transfer out flows from Beijing–Tianjin and the northeast region, (**b**) net carbon transfer out flows from the north coast and central coast regions, (**c**) net carbon transfer out flows from the central and south coast regions, and (**d**) net carbon transfer-out flows from the southwest region.

The spatial distribution of net carbon transfer in the eight regions is shown in the map of China using ArcGIS10.3.1. As shown in Figure 5 below, the dark to light blue area represents the net carbon transfer-in amount from highest to lowest, and the dark to light yellow area represents the net carbon transfer-out amount from highest to lowest. The direction of the arrow represents the transfer direction of the embodied carbon between regions and the thickness represents the amount of embodied carbon.

The map shows that the north mainly inputs embodied carbon to the northwest and the south mainly inputs embodied carbon to the central and north. The eight regions are divided into three categories. The first category is the one-way transfer out region known as northern metropolis, in which the net transfer out of northern metropolis is positive, and its input of embodied carbon to the other seven regions, among which the input to the northern coast region and northwest region is the highest, and inputs to the northeast region and central coast region are lower. The northern metropolis mainly includes the cities of Beijing and Tianjin, which ranked first and third in the country in terms of GDP per capita in 2017 and are economically developed regions. From a sectoral perspective, the main transfer-out sectors are electricity and hot water production, which have the highest carbon emission coefficients. The northern metropolis imports a large number of low value-added and high-carbon-emission products from other regions to meet its economic development needs, and it is highly dependent on interprovincial trade. The second category is a combination of two-way transfer-out and transfer-in amounts, which includes the northern coastal region, the northern coastal region, the northeastern region, the central region, the central coast, the southern coast, and the southwestern region. The net transfer in the central coast, the south coast, and the southwest region is positive. These regions transfer a large amount of embodied carbon to the northwest and northeast regions, but also transfer carbon from the northern metropolis; the amount of carbon transferred out is greater than the amount of carbon transferred in. The net carbon transfer in the northern coastal region, the northeastern region, and the central region is negative. On the one hand, these regions receive the carbon input of Beijing–Tianjin and the southern regions; on the other hand, they also import embodied carbon to the northwest region. However, on the whole, they receive more carbon transfers than other regions, a result of containing more resource-based provinces. Such regions supply most of the goods and services produced to and imported from other regions through interprovincial trade, and interprovincial trade has a large impact on the carbon emissions of such regions. The third category is the one-way transfer region, the northwest region, which has a net carbon transfer from the other seven regions. The northwest region is energy-rich, an important energy base, and exports a large number of carbon-intensive products to other regions. All in all, the regions with positive net carbon transfer, such as the northern metropolitan and central and southern coastal regions, which are in the eastern part of China, import large amounts of energy products from energy-rich regions in order to meet the needs of economic development. These regions can reduce emissions in two ways. One is to transfer the excessive demand for energy-intensive products in the region and the other is to cooperate with other regions through capital or technology to improve energy conservation and emission reduction technologies in other regions. Regions with negative net carbon transfer, such as the north coast and central regions, are geographically linked to the east and west regions. Their industries are often the upstream industries of the eastern region, and they have a strong ability to bear the industrial transfer from the eastern region, both in terms of talent pool and the accumulation of related industrial resources. In the west, a large number of underdeveloped areas in the northwest region are widely distributed and have a strong regional environmental carrying capacity. It can reduce emissions by eliminating outdated production capacity, improving energy efficiency, and adjusting the energy mix.

### 4.3. Carbon Compensation Accounting Results

One approach to regional horizontal carbon compensation is to establish a regional horizontal carbon compensation system with direct economic compensation led by the government. Based on regional carbon transfer accounting, the net carbon transfer-out region (e.g., the urbanized region and the industrially developed region) gives certain economic compensation to the net carbon transfer in the region (e.g., resource-based region) to achieve regional equitable development. In this paper, the compensation amount is calculated based on the net carbon transfer amount, as shown in Table 3.

As seen in Table 2, Guangdong, Zhejiang, Beijing, Henan, Chongqing, Jiangsu, Shaanxi, and Jiangxi have more carbon compensation in 30 provinces (cities and autonomous regions), which are priority areas to pay carbon compensation costs. Beijing, Zhejiang, Henan, Chongqing, and Guangdong are required to compensate eight regional provinces (cities and autonomous regions). Spatially, the provinces that should pay carbon compensation costs are mainly in the central and eastern regions, while the provinces that should receive carbon compensation costs are mainly in the northwestern regions. In addition, economically developed regions generally compensate economically less developed regions.

Specifically, Beijing in the northern metropolis compensates the greatest amount to the northwest, especially to Inner Mongolia, which needs to be compensated for RMB 2.269 billion. Guangdong, one of the most economically developed regions in China, needs to compensate Liaoning (RMB 2.579 billion) in the northeast, Guangxi (RMB 2.393 billion) in the southwest, Inner Mongolia (RMB 2.165 billion) in the northwest, Hebei (RMB 1.768 billion) in the north, and Henan (RMB 1.483 billion) in the central region; its compensation amounts are larger that Beijing’s. Zhejiang and Jiangsu, which are located on the central coast with better economic development, mainly compensate the central region, northwest region, and north coast region, among which Zhejiang mainly compensates Hebei (RMB 1.971 billion) and Shandong (RMB 1.132 billion) in the central coast region, Inner Mongolia (RMB 1.811 billion) in the northwest region, and Jiangsu (1.640 billion) in the central coast region; Jiangsu mainly compensates central Anhui (RMB 1.802 billion), Hebei (RMB 1.072 billion), and Inner Mongolia (RMB 977 million) in the northwestern region; and Jiangsu mainly compensates Anhui (RMB 1.802 billion), Hebei (RMB 1.072 billion), and Inner Mongolia (RMB 977 million) in the central region. Henan, which is the largest overall economy in the central region, also mainly compensates Inner Mongolia in the northwest region (RMB 2.076 billion), Shandong in the central coast region (RMB 1.428 billion), and Shanxi in the central region (RMB 1.205 billion). Jiangxi, another central region, also mainly compensates some provinces in the northwest region, central region, and northern coast. The most compensation to the northwest region is RMB 828 million to Inner Mongolia, RMB 553 million to Shandong on the north coast, and RMB 598 million to Hubei and RMB 520 million to Shanxi in the central region. Heilongjiang, the province in the northeast region with the highest level of required compensation to other regions, compensates RMB 1.287 billion and RMB 586 million to Shanxi in the central region and Inner Mongolia in the northwest region, respectively. Chongqing, the only municipality directly under the central government and the national central city in southwest China, mainly compensates Inner Mongolia in the northwest region and Hebei on the north coast with RMB 1.113 billion and RMB 890 million, respectively. Shaanxi, as the gateway to the northwest, needs to compensate provinces in seven regions, of which the most compensation is needed in the northwest, especially Inner Mongolia, at RMB 1.025 billion, and Sichuan in the southwest, at RMB 812 million. The hot spots of carbon compensation in China are mainly located in Beijing in the north and Shanghai and Guangdong in the eastern coastal region; the cold spots are mainly located in Inner Mongolia and Xinjiang in the northwest region, Shanxi and Hebei in the central region, and Heilongjiang and Jilin in the northeast region.

For specific compensation measures, the transfer of carbon compensation from local to central to local can be achieved by establishing a horizontal transfer system for carbon compensation in different provinces. Both the central government and local governments should set up carbon fund accounts to manage carbon compensation funds. Following the fair compensation principle of “who benefits, who compensates”, the regions with positive net carbon transfer should be the main carbon compensation payers, while the regions with negative net carbon transfer should be the carbon compensation payees. Based on the transaction price of the national carbon market, the amount of carbon compensation will be calculated, and the carbon compensation payer will transfer the carbon compensation funds to the carbon compensation payee. This part of the fund can redistribute inter-provincial abatement costs, provide financial support for carbon abatement in high-carbon-emission regions, and ensure the efficiency of abatement while promoting inter-provincial carbon equity.

## 5. Conclusions and Discussions

In this paper, by accounting for the amount of interprovincial carbon transfer in 2017, we obtained the amount of carbon transfer-out levels, carbon transfer-in levels, and net carbon transfer levels in 30 provinces (municipalities and autonomous regions) in 2017, as well as the specific direction of carbon transfer. At the same time, we divided 30 provinces (municipalities and autonomous regions) into eight regions and analyzed the state of carbon transfer between different regions. Finally, based on the amount of carbon transfer from each province (municipalities and autonomous regions), combined with the carbon price in the national carbon market, the interprovincial carbon compensation amount is accounted for. The main conclusions are as follows. (1) From a provincial perspective, the province with the highest carbon transfer-out amount is Guangdong Province, and the province with the highest carbon transfer-in amount is Inner Mongolia; the provinces with higher carbon transfer-out amounts are mostly economically developed regions, and the provinces with more carbon transfer-in amounts are mostly resource-based. From a sectoral perspective, energy, metallurgy, nonmetal products, coal mining, petroleum, and transportation sectors are the main sectors of carbon transfer. (2) In terms of the spatial distribution of net carbon transfer, most of the net-carbon-importing regions are located in the north, with a small number in the south, while most of the net-carbon-transferring regions are located in Beijing, Tianjin, as well as central and eastern coastal areas. (3) From analyzing the net carbon transfer of the eight regions, it can be seen that the regions with positive net carbon transfer, such as the Beijing–Tianjin and central and south coast regions, ensure their realization of carbon emission reduction tasks by transferring some high-pollution and high-energy-consuming industries to other regions with rapid economic growth; the regions with negative net carbon transfer, such as the northern coastal and central regions, which are geographically linked to the east and west regions, have a strong ability to bear the related industrial transfer in the east, and they also bear the carbon emission pressure from the northwest region. (4) By determining the amount of regional horizontal carbon compensation through the market price method, we can see that the overall spatial pattern of the carbon compensation amount decreases from the center to the periphery in the eastern coastal region, Beijing and Tianjin, and the northern coastal region, showing that the more economically developed regions compensate for the less economically developed regions.

In general, some results of this study are fundamentally consistent with those of previous studies. The previous research based on the data of 2010 [27,29,30] and 2012 [25,26,28] shows that the inter-provincial embodied carbon in China has shifted from economically developed provinces to less developed provinces. This study uses data from 2017 for calculation purposes and shows that the overall distribution of embodied carbon transfer between regions still presents a spatial pattern of transfer from south to north and from east to west. The results confirm that after a period of development, the phenomenon of “carbon leakage” still exists among regions in China [5,36,59]. In contrast to other papers, in addition to using the latest data to track the amount and direction of embodied carbon transfer (transfer in and out) between provinces in China, this study also conducted in-depth research from three aspects. First, based on the characteristics of carbon transfer, the key sectors of carbon reduction in different provinces were identified and targeted suggestions were proposed. Second, by increasing the perspective of spatial transfer in eight regions, we can reflect the embodied carbon transfer between different regions in China more accurately and reveal the differences in economic development, energy consumption, and industrial structure between regions. Furthermore, we put forward a specific accounting scheme for regional horizontal carbon compensation to promote carbon equity among regions.

This study has several limitations. First, because it is difficult to obtain continuous inter-provincial input–output data, this study only uses data from 2017 for static analysis, and fails to analyze the time series of each province. Second, this study lacks an exploration of the dynamic driving mechanism of carbon transfer and cannot deeply analyze the complex reasons for the long-term existence of “carbon leakage” between provinces. Therefore, in future research, the inter-provincial input–output time series database can be constructed to analyze the spatiotemporal evolution process of implied carbon transfer. Furthermore, we will further analyze the driving factors behind this process, as well as the impact of dynamic changes among driving factors on the spatial and temporal pattern of carbon transfer. This is of great significance to scientifically and accurately reveal the dynamic mechanism of the formation and evolution of inter-regional carbon transfer.

Based on the analysis and discussion of the results of inter-provincial carbon transfer and carbon compensation, this paper proposes three policies to promote regional carbon reduction. (1) Carbon emissions are a subsidiary product of economic and social development and show great variability among regions with the level of economic development. Each region should formulate appropriate emission reduction policies according to the characteristics of trade carbon transfer. Regions with net carbon transfer-in levels are resource-intensive regions. Based on the resource endowment, energy structure, and industrial structure, they can focus on the key carbon emission sectors and make their own products more low-carbon and environmentally competitive. Regions with net carbon transfer-out levels are economically developed regions with developed service industries which need to rely on imported energy products to meet their own production and consumption needs; these provinces can focus on the source of imported products, changing from high-emission-intensity regions to low-emission-intensity regions without affecting social and economic development and industrial structure. (2) Carbon is a by-product of economic activities on the ecological environment, and is highly correlated with industrial structure. From the distribution of regional trade represented by carbon emission sectors, including the energy sector, metal and nonmetal account for a large share of trade embodied carbon emissions and show the characteristics of transferring from the coast to the middle west. Therefore, adjusting the industrial structure and upgrading technology is the key to emission reduction, especially for the adjustment of the energy industry, which can focus on improving the energy structure and optimizing industrial energy utilization efficiency. On the one hand, they should eliminate backward production capacity or carry out transformation, and promote innovation in green and low-carbon technologies. On the other hand, based on their own energy resource, they should promote clean energy development, such as hydropower and geothermal power, and adjust the energy production structure. (3) According to the principle of “who benefits, who compensates”, an interprovincial compensation mechanism should be established. Based on the net carbon transfer, the economically developed regions should give financial or technical compensation and support to the less developed regions. This will help reduce the pressure of net carbon transfer in regions and promote interprovincial carbon equity.

## Figures and Tables

**Table 1:** The multi-regional input–output table short form. Source: the authors.

		Output	Intermediate Use							Final Use			Exports	Total Output
			Region 1			...	Region m			Region 1	...	Region m		
Input			Sector 1	...	Sector n		Sector 1	...	Sector n					
**Intermediate input**	Region 1	Sector 1	$X_{11}^{11}$	…	$X_{1n}^{11}$	…	$X_{11}^{1m}$	…	$X_{1n}^{1m}$	$Y_{1}^{11}$	…	$Y_{1}^{1m}$	$EX_{1}^{1}$	$X_{1}^{1}$
		…	…	…	…	…	…	…	…	…	…	…	…	…
		Sector n	$X_{n1}^{11}$	…	$X_{nn}^{11}$	…	$X_{n1}^{1m}$	…	$X_{nn}^{1m}$	$Y_{n}^{11}$	…	$Y_{n}^{1m}$	$EX_{n}^{1}$	$X_{n}^{1}$
	⋮	⋮	⋮			⋮	⋮			⋮	⋮	⋮	⋮	⋮
	Region m	Sector 1	$X_{11}^{m1}$	…	$X_{1n}^{m1}$	…	$X_{11}^{mm}$	…	$X_{1n}^{mm}$	$Y_{1}^{m1}$	…	$Y_{1}^{mm}$	$EX_{1}^{m}$	$X_{1}^{m}$
		…	…	…	…	…	…	…	…	…	…	…	…	…
		Sector n	$X_{n1}^{m1}$	…	$X_{nn}^{m1}$	…	$X_{n1}^{mm}$	…	$X_{nn}^{mm}$	$Y_{n}^{m1}$	…	$Y_{n}^{mm}$	$EX_{n}^{m}$	$X_{n}^{m}$
**Import**			$IM_{1}^{1}$	…	$IM_{n}^{1}$	…	$IM_{1}^{m}$	…	$IM_{n}^{m}$	$YIM_{1}^{1}$	…	$YIM_{1}^{m}$		
**Value added**			$V_{1}^{1}$		$V_{n}^{1}$		$V_{1}^{m}$		$V_{n}^{m}$					
**Total input**			$X_{1}^{1}$		$X_{n}^{1}$		$X_{1}^{m}$		$X_{n}^{m}$					

**Table 2:** Classification and net carbon transfer for each major region.

Eight Regions	Provinces	Net Carbon Transfer (Mt)
Beijing–Tianjin	Beijing and Tianjin	145.23
North Coast	Hebei and Shandong	−544.23
Northeast	Heilongjiang, Jilin, and Liaoning	−47.86
Central Coast	Jiangsu, Shanghai, and Zhejiang	347.32
South Coast	Fujian, Guangdong, and Hainan	340.52
Central	Shanxi, Henan, Anhui, Hubei, Hunan, and Jiangxi	−196.49
Northwest	Inner Mongolia, Shaanxi, Ningxia, Gansu, Qinghai, and Xinjiang	−337.62
Southwest	Sichuan, Chongqing, Yunnan, Guizhou, and Guangxi	293.13

**Table 3:** Top eight provinces in terms of the carbon compensation amount.

Provinces Offering Compensation	Provinces Receiving Compensation	Affiliated Region	Amount (Million RMB)	Provinces Offering Compensation	Provinces Receiving Compensation	Affiliated Region	Amount (Million RMB)
**Beijing**	Inner Mongolia	Northwest	22.69	**Guangdong**	Inner Mongolia	Northwest	21.65
	Xinjiang		9.51		Xinjiang		9.79
	Shaanxi		4.96		Ningxia		5.78
	Ningxia		4.91		Shaanxi		4.58
	Gansu		2.11		Gansu		4.12
	Qinghai		0.01		Qinghai		0.28
	Hebei	North Coast	15.97		Henan	Central	14.83
	Shandong		13.39		Shanxi		9.54
	Jilin	Northeast	2.92		Jiangxi		4.94
	Heilongjiang		8.88		Hunan		4.64
	Liaoning		7.43		Anhui		4.43
	Shanxi	Central	9.45		Hubei		2.25
	Anhui		2.97		Liaoning	Northeast	25.79
	Henan		2.13		Heilongjiang		5.53
	Hubei		1.35		Jilin		4.94
	Jiangxi		0.98		Guangxi	Southwest	23.93
	Jiangsu	Central Coast	7.3		Guangxi		7.55
	Zhejiang		5.74		Sichuan		1.14
	Shanghai		2.62		Yunnan		0.55
	Fujian	South Coast	3.41		Hebei	North Coast	17.68
	Guangdong		3.09		Shandong		13.95
	Hainan		0.38		Jiangsu	Central Coast	11.8
	Guangxi	Southwest	3.39		Shanghai		3.66
	Sichuan		1.86		Fujian	South Coast	5.89
	Guangxi		0.66		Hainan		0.35
	Chongqing		0.52		Tianjin	Beijing–Tianjin	1.13
	Yunnan		0.41	**Zhejiang**	Inner Mongolia	Northwest	18.11
	Tianjin	Beijing–Tianjin	2.8		Xinjiang		5.01
**Chongqing**	Inner Mongolia	Northwest	11.13		Ningxia		3.85
	Xinjiang		5.26		Shaanxi		3.59
	Shaanxi		3.94		Gansu		3.03
	Ningxia		3.33		Qinghai		0.77
	Gansu		1.25		Hebei	North Coast	19.71
	Qinghai		0.34		Shandong		11.32
	Henan	Central	6.17		Shanxi	Central	11.48
	Shanxi		5.43		Jiangxi		4.83
	Anhui		1.79		Anhui		4.69
	Jiangxi		1.71		Henan		4.14
	Hunan		1.45		Hunan		3.04
	Hubei		0.45		Hubei		1.37
	Hebei	North Coast	8.9		Liaoning	Northeast	8.36
	Shandong		6.55		Jilin		5.46
	Heilongjiang	Northeast	3.92		Heilongjiang		3.13
	Liaoning		3.85		Jiangsu	Central Coast	16.4
	Jilin		1.15		Guangxi	Southwest	3.53
	Guangxi	Southwest	3.81		Guangxi		3.48
	Yunnan		0.69		Yunnan		1.72
	Jiangsu	Central Coast	3.28		Sichuan		0.72
	Shanghai		1.05		Chongqing		0.02
	Fujian	South Coast	1.96		Fujian	South Coast	2.98
	Guangdong		0.85		Guangdong		1.34
	Tianjin	Beijing–Tianjin	0.65		Hainan		0.08
**Jiangsu**	Anhui	Central	18.02		Tianjin	Beijing–Tianjin	2.28
	Shanxi		11.33	**Shaanxi**	Inner Mongolia	Northwest	10.25
	Hunan		1.72		Xinjiang		6.17
	Jiangxi		0.7		Gansu		2.99
	Hubei		0.21		Ningxia		2.2
	Inner Mongolia	Northwest	9.77		Qinghai		0.13
	Xinjiang		2.87		Sichuan	Southwest	8.12
	Ningxia		2.02		Guangxi		1.26
	Gansu		1.1		Guangxi		0.46
	Qinghai		0.44		Hainan	South Coast	5.4
	Hebei	North Coast	10.72		Fujian		1.99
	Shandong		6.58		Liaoning	Northeast	2.73
	Liaoning	Northeast	2.9		Heilongjiang		1.98
	Heilongjiang		2.08		Jilin		1.87
	Jilin		1.98		Shanxi	Central	5.34
	Shanghai	Central Coast	2.86		Anhui		1.09
	Fujian	South Coast	0.54		Hebei	North Coast	2.33
	Guangxi	Southwest	0.15		Jiangsu	Central Coast	0.77
**Henan**	Inner Mongolia	Northwest	20.76	**Jiangxi**	Inner Mongolia	Northwest	8.28
	Xinjiang		10.06		Ningxia		2.18
	Ningxia		5.37		Xinjiang		1.67
	Shaanxi		2.45		Shaanxi		0.84
	Gansu		2.07		Gansu		0.75
	Qinghai		0.16		Qinghai		0.08
	Shandong	North Coast	14.28		Hubei	Central	5.98
	Hebei		8.68		Shanxi		5.2
	Liaoning	Northeast	7.88		Henan		0.74
	Heilongjiang		6.66		Hunan		0.63
	Jilin		0.29		Shandong	North Coast	5.53
	Shanxi	Central	12.05		Hebei		2.76
	Anhui		0.99		Heilongjiang	Northeast	3.26
	Hubei		0.95		Liaoning		1.23
	Fujian	South Coast	2.78		Jilin		0.42
	Jiangsu	Central Coast	2.15		Fujian	South Coast	1.69
	Guangxi	Southwest	0.57		Guangxi	Southwest	0.85
	Sichuan		0.34		Guangxi		0.37
	Tianjin	Beijing–Tianjin	0.18		Sichuan		0.19

## Data Availability

The Chinese multiregional input–output data and CO_2_ emission factors are selected from the China Carbon Accounting Database (CEAD) (https://www.ceads.net.cn/ (accessed on 15 December 2022)).
